# Self-Standing Mo/MoO_2_ Porous Flake Arrays for Efficient Hydrogen Evolution Reaction in High-pH Media

**DOI:** 10.1021/acsami.4c14140

**Published:** 2024-10-07

**Authors:** Chuanyong Jian, Jiashuai Yuan, Qian Cai, Wenting Hong, Wei Liu

**Affiliations:** † CAS Key Laboratory of Design and Assembly of Functional Nanostructures, Fujian Provincial Key Laboratory of Nanomaterials, Fujian Institute of Research on the Structure of Matter, Chinese Academy of Sciences, Fuzhou 350002, China; ‡ College of Chemistry and Materials, Fujian Normal University, Fuzhou, Fujian 350007, China

**Keywords:** Mo/MoO_2_, local acidic environment, porous flake array structure, high current density, alkaline hydrogen evolution reaction

## Abstract

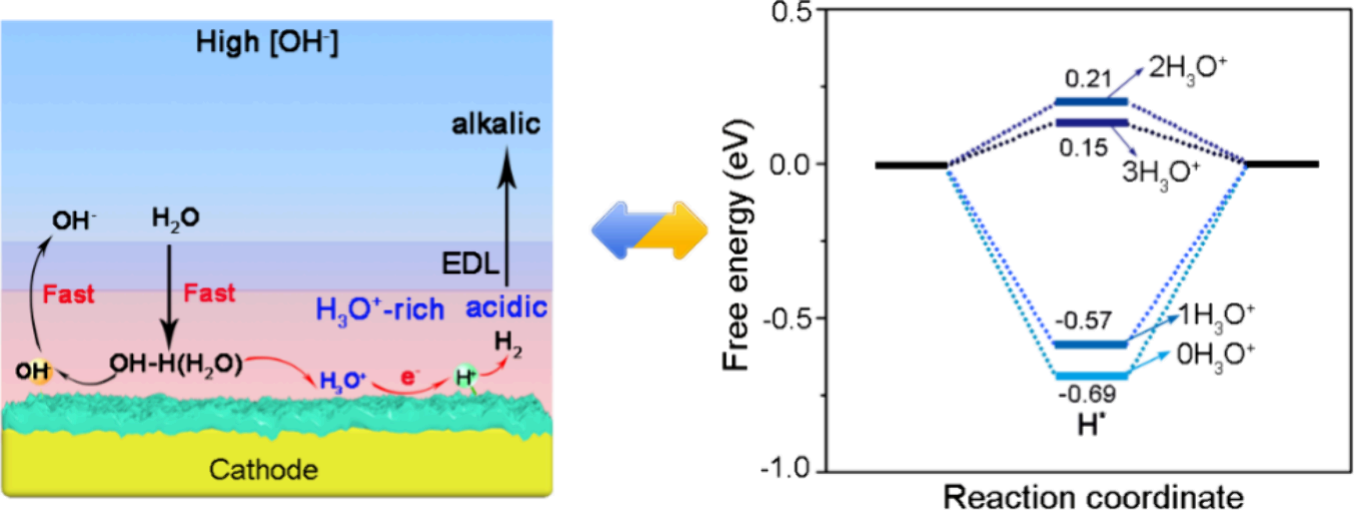

The alkaline hydrogen evolution reaction (HER) is limited by scarce proton availability, resulting in slower reaction kinetics compared to those under acidic conditions. Enhancing the local chemical environment of protons on the catalyst surface can improve the intrinsic reaction kinetics. Here, we design a Mo/MoO_2_ metallic heterojunction that creates an acidic-like environment with a proton-rich surface, significantly enhancing HER performance in alkaline electrolytes, as confirmed by in situ spectroscopy and electrochemical analysis. A self-standing Mo/MoO_2_ catalytic electrode is fabricated via a controlled pyrolysis-reduction strategy. This electrode exhibits exceptional HER activity, with low overpotentials of 65 mV at 10 mA cm^–2^ and 315 mV at 500 mA cm^–2^, a Tafel slope of 38.2 mV dec^–1^, and stability exceeding 60 h at −300 mA cm^–2^ in alkaline solution. The porous flake array structure of the Mo/MoO_2_ heterojunctions enhances the adjacent hydronium (H_3_O^+^) concentration, resulting in a Δ*G*_H*_ value of 0.15 eV and a water dissociation energy barrier of 0.37 eV in an alkaline medium. The successful preparation of a large-area electrode (2 cm × 2 cm) demonstrates the scalability of this approach for fabricating molybdenum-based catalytic electrodes with enhanced HER activity in alkaline environments.

## 1 Introduction

Hydrogen (H_2_), known for its high energy density and emission-free nature, serves as an eco-friendly alternative to conventional fossil fuels.^1−4^ The electrocatalytic hydrogen evolution reaction (HER) powered by solar technologies or wind energy for scalable hydrogen production is considered a sustainable strategy.^5−8^ Recently, electrocatalytic HER in alkaline media has garnered increased attention due to the durability of electrode materials, long operating life, and the affordability of electrolyzer setups, resulting in cost and safety concerns in practical applications.^9−11^ In neutral or alkaline environments, HER kinetics initiate with water dissociation (H_2_O + ∗ + e^–^ → H* + OH^–^, where ∗ denotes the active site) and proceed with either Heyrovsky (H* + H_2_O + e^–^ → H_2_ + OH^–^) or Tafel reactions (H* + H* → H_2_).^12,13^ Compared with HER in an acidic environment, the H_2_O dissociation step to produce available protons can be added in neutral/alkaline medium, resulting in a low concentration of hydrogen (H*) on or near the surface of the catalyst. Currently, noble metals are favored as HER catalysts, yet their scarcity and high cost restrict widespread commercial use.^14−16^ Furthermore, the HER activity of platinum-based catalysts in high-pH environments (e.g., 1 M KOH) is significantly lower—often by 2 to 3 orders of magnitude—compared to acidic conditions, possibly due to the pH influence on H_2_O dissociation and/or H* adsorption energy barrier.^17^ Therefore, developing affordable, efficient, and stable non-noble-metal catalysts for HER under high-pH conditions remains a critical challenge for economical hydrogen production through electrolysis.

Among various materials, Mo-based materials are highly regarded as efficient HER electrocatalysts owing to their natural chemical and electronic configurations, excellent electrical properties, and robust mechanical characteristics.^18^ Efforts to boost the catalytic efficiency of these materials have included extensive research aimed at uncovering the active sites of the catalysts by augmenting their specific surface area, thereby improving the kinetics of alkaline HER. For example, 3D macroporous framework MoS_2_–Mo_2_C supported on Mo plate, Ni, and MoO_3_ nanoparticle anchored NiO nanosheets, and the hierarchical NiMoO_*x*_/NiMoS heterostructure array has been prepared for the excellent HER electrocatalysts.^19−21^ Apart from the active sites, some strategies are mainly dedicated to facilitating intrinsic HER activity by ameliorating the catalyst materials through various methods,^22−27^ including doping heteroatoms, constructing heterostructure, engineering strain, and introducing oxyphilic active elements. For instance, P-doped MoO_2_/MoS_2_, 1T_0.63_-MoSe_2_@MoP multiphase-interface heterostructure, and unique distorted IrO_2_ structure on V_2_O_5_ support have been systematically explored for the enhanced alkaline HER activities. While the above strategies can modulate their electronic structure and certain reaction steps of intermediates to accelerate HER rate, these still cannot escape the pH-dependent kinetics of HER process, leading to a lack of substantial advancements in alkaline electrolytes. Additionally, the local chemical environment at or near the catalytic surface significantly influences the reaction pathway and HER kinetics.^28−30^ Recently, the reported Ir–H_*x*_WO_3_ and Pt_tet_@Ni(OH)_2_ catalysts create a favorable local chemical environment on or near the catalytic surface to result in acidic-like HER kinetics in high-pH environments, thereby improving alkaline HER activity.^31,32^ Therefore, integrating the design of electrocatalytic active sites with modulation of the local chemical environment to achieve acidic-like HER kinetics is essential for developing highly efficient electrocatalysts in nonacidic electrolytes.

Herein, we construct three-dimensional (3D) Mo/MoO_2_ heterojunction flake arrays to create a local acidic-like environment with strong abilities of proton generation and greatly boost HER performance in alkaline medium. Porous Mo/MoO_2_ flake arrays on the Mo plate are fabricated via a controlled pyrolysis-reduction strategy. During in situ pyrolysis processes, a Mo/MoO_2_ heterojunction could be formed, which exhibits an acidic-like HER activity with a low overpotential of 65 mV at 10 mA cm^–2^ and a Tafel slope of 38.2 mV dec^–1^ in alkaline electrolyte. Moreover, the as-prepared Mo/MoO_2_ metallic heterojunction requires only a small overpotential of 315 mV to achieve a high current density of −500 mA cm^–2^ and demonstrates exceptional catalytic stability, maintaining performance at −300 mA cm^–2^ for over 60 h in alkaline solution, comparable to that in acidic environments. Density functional theory (DFT) calculations reveal that the Mo/MoO_2_ heterojunction has a Δ*G*_H*_ value of 0.15 eV by increasing adjacent H_3_O^+^ concentration and an energy barrier of 0.37 eV for H_2_O dissociation in alkaline medium. This work will provide inspiration for tailoring a local chemical environment to improve alkaline HER kinetics and pathway.

## 2 Results and Discussion

**Figure 1:**
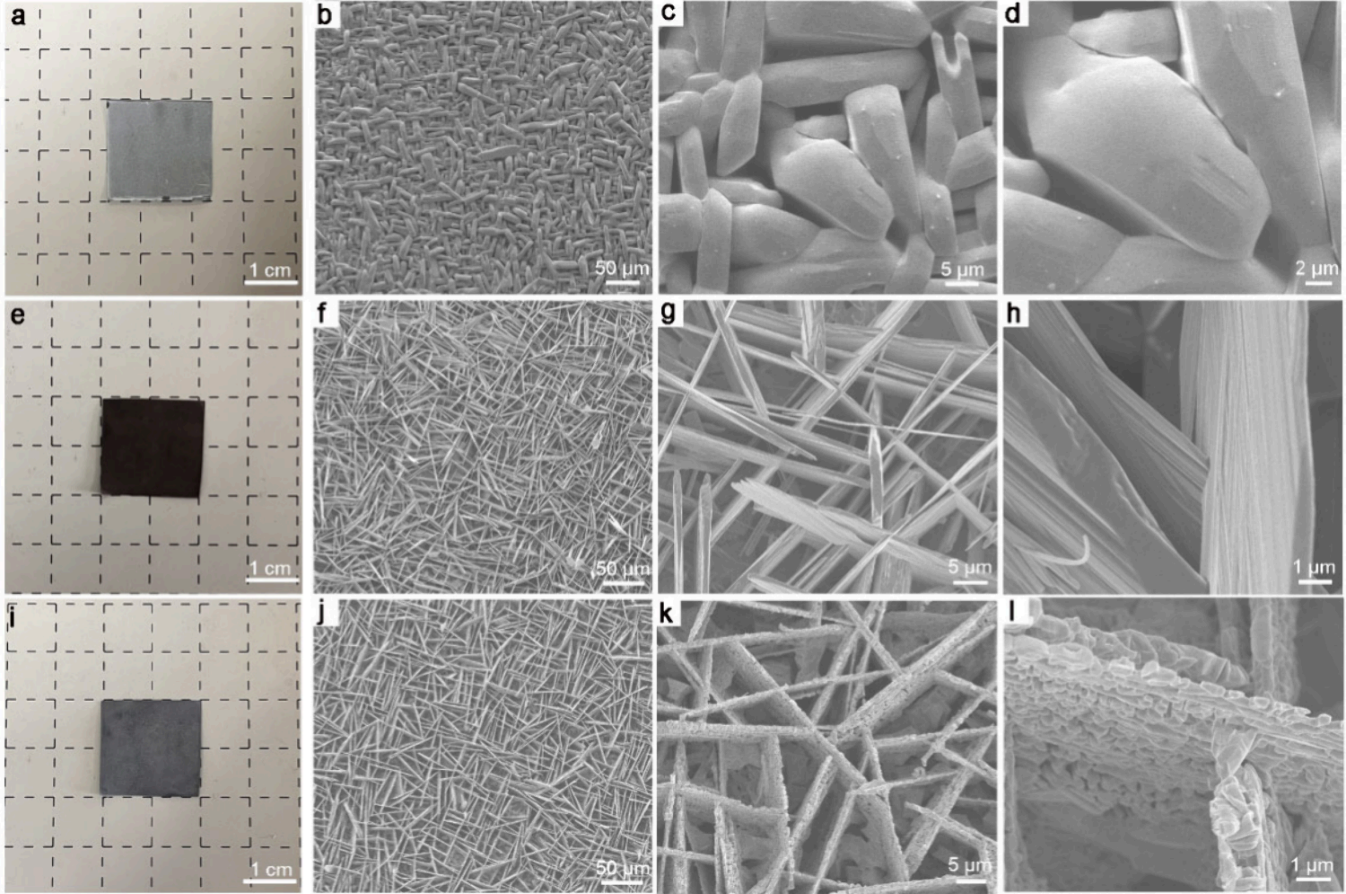
Photographs and SEM images of (a–d) vertical MoO_3_ prisms, (e–h) MoO_*x*_ flake arrays, and (i–l) porous Mo/MoO_2_ flake arrays on a Mo plate.

**Figure 2:**
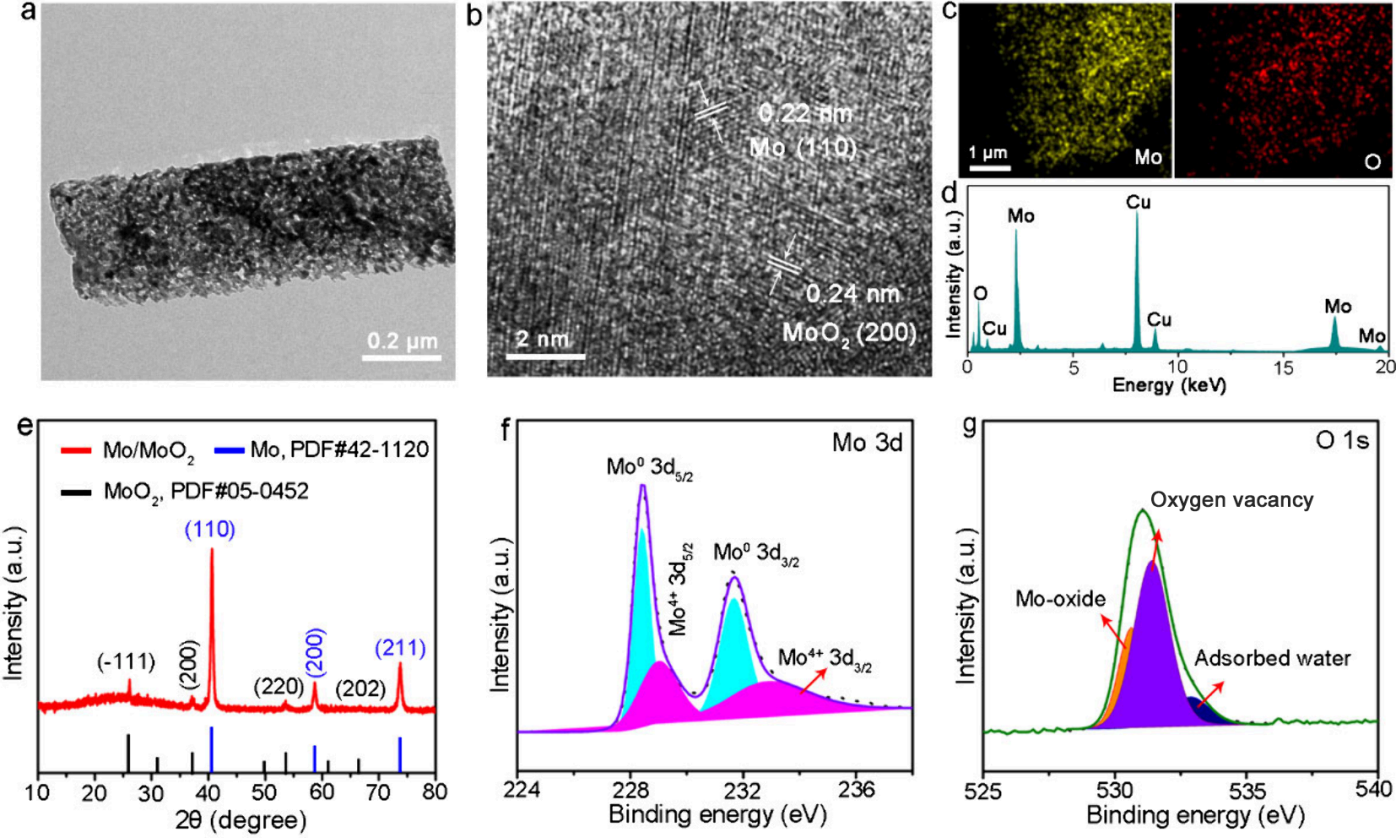
(a, b) Low- and high-resolution TEM images of the Mo/MoO_2_ catalyst. (c) TEM-EDS mapping of Mo/MoO_2_ showing the uniform distribution of the O (red) and Mo (yellow) elements. (d) EDS spectra were obtained for elemental analysis of Mo/MoO_2_. (e) XRD pattern of the Mo/MoO_2_ catalyst. High-resolution XPS spectra of (f) Mo 3d and (g) O 1s.

The synthetic procedure of the porous Mo/MoO_2_ flake arrays is prepared via a series of thermal redox reaction (Figure S1). In brief, prism-shaped MoO_3_ crystals, with a thickness ranging from approximately 2.5 to 5 μm, are directly grown on the surface of the Mo plate in air at 650 °C (Figure 1a–d and Figure S2). Subsequent pyrolysis at 800 °C under an Ar atmosphere converted the as-prepared MoO_3_ prisms into MoO_*x*_ (MoO_2_ + Mo_4_O_11_) flake arrays (Figure S3). Finally, the desired Mo/MoO_2_ metallic heterojunction is obtained by pyrolyzing the free-standing MoO_*x*_ precursor at 700 °C in an Ar/H_2_ (v/v = 10:1) atmosphere. The reaction mechanism can be explained by using the theoretical gas–solid reaction model, incorporating the shrinking core model (SCM) and the cracking core model (CCM) (Figure S4). These synthesis steps are performed under gentle conditions, are scalable, and enable the production of large-area Mo/MoO_2_ catalytic electrodes (Figure 1i). Scanning electron microscopy (SEM) is used to analyze the morphology and structure of the vertically aligned MoO_*x*_ and Mo/MoO_2_ flake arrays. In Figure 1e–h and Figure S5, the as-grown MoO_*x*_ flakes are normal to the Mo plate. These flakes show a smooth cuboid-like shape with an average thickness of 1–2 μm as well as a height and bottom length of 3–5 and 10–20 μm, respectively (Figure 1g,h). After the annealing of MoO_*x*_ flakes in a mixture of Ar/H_2_ atmosphere, these smooth flakes are transformed into a porous structure with some large channels and small nanopores (Figure 1i–l and Figure S5d–f). Their morphologies (cuboid-like shape) and dimensions (an average thickness is 1–2 μm, a height and bottom length of 3–5 and 10–20 μm) are almost unchanged compared to the corresponding vertical MoO_*x*_ flakes. This structure effectively pumps the liquid-phase electrolyte to the catalyst surface through significant capillary action. As a result, it reduces the adhesion at the gas–solid interface, promoting the release of hydrogen bubbles from the catalyst surface, which is essential for efficient HER at high current densities. Additionally, the porous flake arrays structure provides a plethora of exposed catalytic active edge sites. Low-magnification transmission electron microscopy (TEM) images, depicted in Figure 2a and Figure S6, show typical porous flakes with interconnected channels featuring concave and convex shapes. Figure 2b reveals a high density of nanoparticles smaller than 2 nm emerging from molybdenum-based flakes, with two sets of lattice fringes and spacings of 0.22 and 0.24 nm observed under high-magnification TEM images. These correspond to the (200) and (002) facets of Mo nanoparticles and the MoO_2_ matrix, respectively. Noticeably, TEM-EDS mapping confirms that the Mo and O elements are uniformly distributed throughout the crystal (Figure 2c), and the chemical stoichiometry is determined to be 2:1 based on the EDS spectrum analysis (Figure 2d). Therefore, drawing on the described crystal structure and morphological characterizations, Mo/MoO_2_ heterojunctions are indeed constructed. The crystal structure of the Mo/MoO_2_ catalyst is further verified by X-ray diffraction (XRD) pattern (Figure 2e and Figure S7). There are three obvious characteristic diffraction peaks of 41.1°, 59.3°, and 74.2°, which can be ascribed to Mo^0^ (PDF#42-1120). Meanwhile, the other peaks of Mo/MoO_2_ catalyst located at 2θ ≈ 26.3°, 37.0°, 53.7°, and 66.9° can be indexed as the peaks of metallic MoO_2_ (PDF#05-0452), which indicates that Mo^0^ and MoO_2_ phases are successfully thermal reduction synthesized. X-ray photoelectron spectroscopy (XPS) provides deeper insight into the chemical states of the Mo/MoO_2_ catalyst. As illustrated in Figure 2f, two sets of peaks are observed at 228.1 and 231.3 eV and at 229.2 and 232.6 eV, which correspond to Mo^0^ and Mo^4+^, respectively. Furthermore, the O 1s spectrum can be deconvoluted to reveal contributions from metal–oxygen bonds as well as surface-adsorbed oxygen, as shown in Figure 2g.

**Figure 3:**
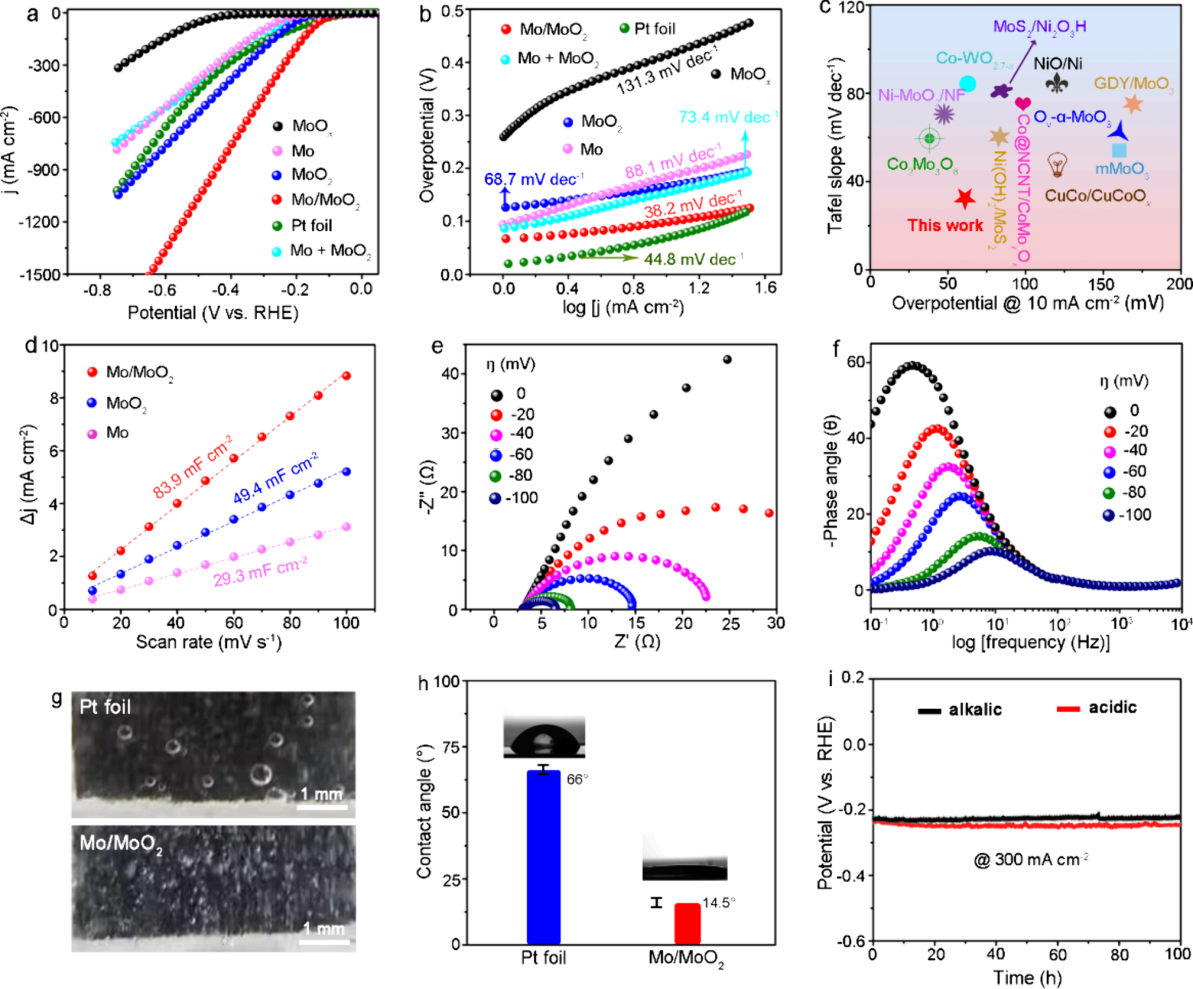
(a) Polarization (LSV) curves of MoO_2_, Mo, Mo/MoO_2_, Mo + MoO_2_, and Pt foil catalysts in 1.0 M KOH solution. (b) Corresponding Tafel plots. (c) Comparison of overpotentials (at 10 mA cm^–2^) and Tafel slopes between Mo/MoO_2_ and previously reported high-performance HER catalysts in alkaline electrolytes. The relevant references for these electrocatalysts are given in Table S1. (d) Determination of *C*_dl_ by plotting the change in current density (Δ*j*) against scan rates (10–100 mV s^–1^). (e) Nyquist plots and (f) corresponding Bode phase plots of the Mo/MoO_2_ catalyst under increasing overpotentials. (g) H_2_ bubbles desorption behaviors of Mo/MoO_2_ and commercial Pt foli at 100 mA cm^–2^. (h) Photos showing a stark contrast in the release of H_2_ bubbles on Pt foil and Mo/MoO_2_ surfaces. (i) Chronopotentiometry measurements recorded on Mo/MoO_2_ at current densities of ∼300 mA cm^–2^ for 100 h in both media.

The electrocatalytic HER performances of the catalysts are assessed using a three-electrode setup in H_2_-saturated 1.0 M KOH at room temperature. Linear sweep voltammetry (LSV) curves were recorded at a scanning rate of 5 mV s^–1^ for various catalytic electrodes with *iR* compensation. The potential required to achieve an HER current density of −10 mA cm^–2^ is considered to be a key parameter for evaluating HER activity. As shown in Figure 3a, the Mo/MoO_2_ catalyst exhibits a low overpotential of 65 mV at 10 mA cm^–2^, outperforming MoO_2_ (132 mV) and Mo (163 mV). For practical applications, a high-current density HER performance is crucial. Therefore, we further investigate the HER performance at a high current density in alkaline conditions. Mo/MoO_2_ catalyst shows the overpotentials of 315 mV at 500 mA cm^–2^, which are much smaller than that of Pt foil (397 mV at 500 mA cm^–2^) electrodes. Additionally, the physical mixture of Mo and MoO_2_ (Mo + MoO_2_) shows a lower overpotential compared to the Mo catalyst at current densities below 326 mA cm^–2^. The significant improvement in HER performance can be attributed to the synergistic effects of MoO_2_ and Mo components in facilitating H_2_O dissociation and H* desorption during the alkaline HER process. However, the alkaline HER performance of Mo + MoO_2_ still falls short of the Mo/MoO_2_ catalyst, indicating that the Mo/MoO_2_ interface formed through chemical bonds can intrinsically enhance the alkaline HER performance. To better understand the underlying reaction kinetics of HER on the Mo/MoO_2_ catalyst surface, Tafel plots (Figure 3b) are analyzed under alkaline conditions. The Mo/MoO_2_ catalyst exhibits a significantly reduced Tafel slope (38.2 mV dec^–1^) compared to those of Mo (88.1 mV dec^–1^) and MoO_2_ (68.7 mV dec^–1^), which is even lower than the commercial Pt foil (44.8 mV dec^–1^). This suggests that the energy barrier for additional H_2_O dissociation has been greatly lowered, and hydrogen generation occurs with optimal kinetics via the Tafel pathway due to the proton-rich environment on the Mo/MoO_2_ surface. The HER performances of MoO_2_, Mo, and Mo/MoO_2_ powders are further tested using the rotating disk electrode method in a 1.0 M KOH electrolyte (Figure S8a). Even at high current densities, the Mo/MoO_2_ powders continue to display superior HER activity, with a low overpotential of −331 mV at −500 mA cm^–2^, outperforming Mo and MoO_2_ and surpassing those of many previously reported metal oxides (Figure 3c).

**Figure 4:**
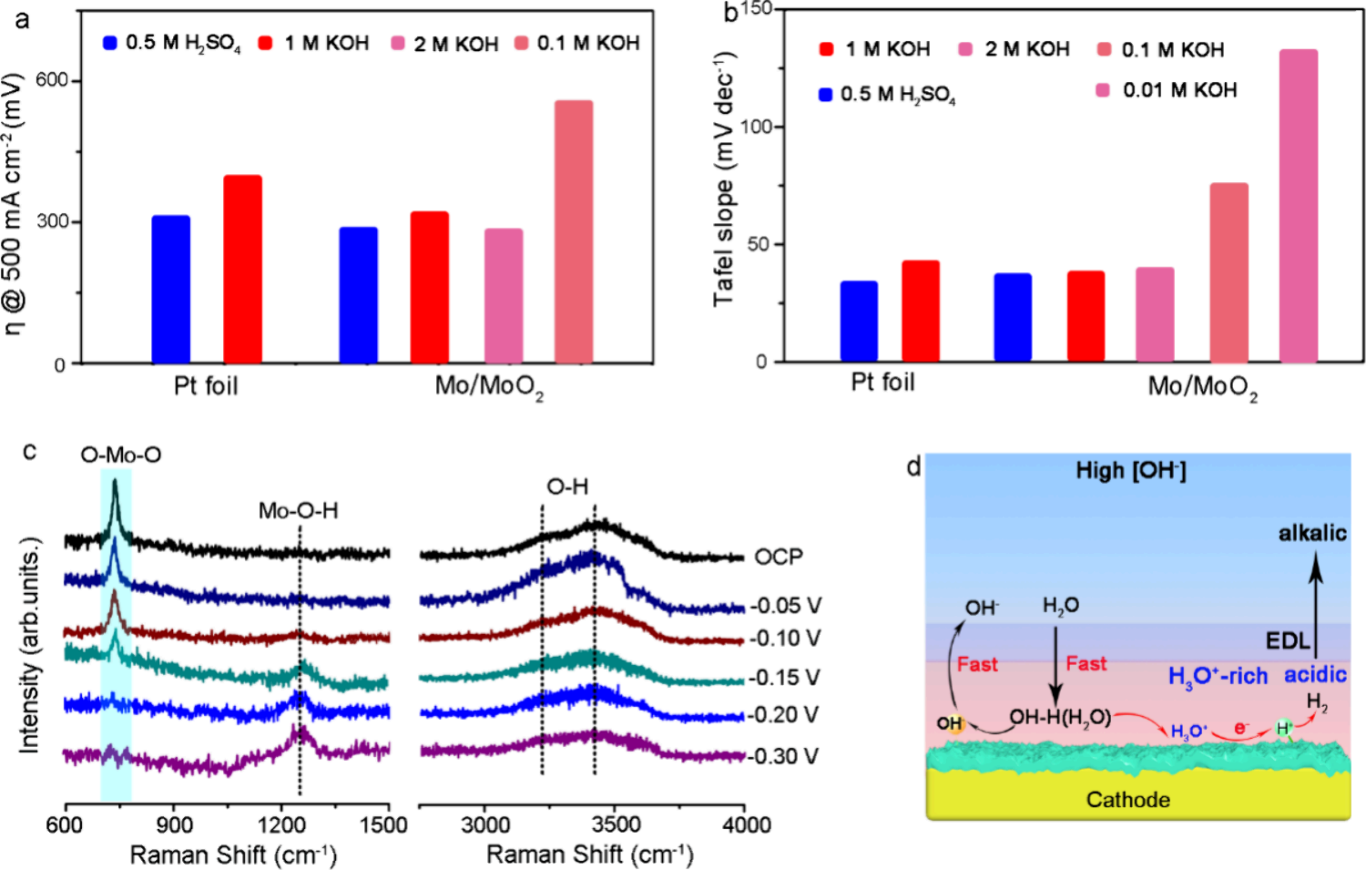
(a) Overpotentials at 500 mA cm^–2^ for a Pt foil and Mo/MoO_2_ in acidic (0.5 M H_2_SO_4_) and different alkaline media (0.01, 0.1, 1.0, and 2.0 M KOH). (b) Corresponding Tafel plots. (c) Operando electrochemical Raman spectra of Mo/MoO_2_ from the OCP to −0.3 V_RHE_. (d) Schematic diagram of the water reduction mechanism on the Mo/MoO_2_ cathode in solutions with high [OH^–^].

To achieve a deeper understanding of the interfacial charge-transfer kinetics in the HER process, the double-layer capacitance (*C*_dl_) and electrochemical impedance spectroscopy (EIS) of the Mo/MoO_2_ catalyst were studied, using Mo and MoO_2_ as reference points. The Mo/MoO_2_ catalyst exhibits a high *C*_dl_ value of 83.9 mF cm^–2^, demonstrating that the Mo/MoO_2_ interfaces act as accessible active sites for the adsorption of H_2_O reactants and intermediates during the alkaline HER process (Figure 3d and Figure S8b–d). Operando EIS was performed at various voltages to gain insights into proton accumulation from H_2_O activation on the Mo/MoO_2_ catalyst surface. As shown in Figure S9a, the equivalent circuit consists of three parts:^33,34^ (1) electrolyte resistance (*R*_s_); (2) interface reaction charge transfer (CPE and *R*_1_), where lower *R*_1_ values indicate faster charge transfer kinetics for H_2_O molecule adsorption and activation at low frequencies; (3) relaxation behavior of charge associated with reaction intermediates (H* and OH*) at high frequencies (*C*_ϕ_ and *R*_2_). As expected, the Mo/MoO_2_ catalyst shows a similar *R*_s_ value (∼2.6 Ω) under the applied potentials from 0 to −100 mV (Figure 3e and Table S2). The Bode plots attribute the responses in the low-frequency region to interfacial charge transfer and those in the high-frequency region to inner-layer electron transport within the catalyst.^35^ The rapid decrease in the phase angle peak in the low-frequency region for Mo/MoO_2_ suggests enhanced charge transfer kinetics at the catalyst–H_2_O interface (Figure 3f). Moreover, *R*_1_ can sharply decrease to 3.1 Ω with a small H_2_O diffusion resistance at an applied overpotential of −40 mV, suggesting the Mo/MoO_2_ catalyst surface is favorable for the adsorption and activation of H_2_O molecules under low overpotentials. Notably, increasing the applied overpotentials leads to significantly higher *C*_ϕ_ values and lower *R*_2_ values. At −60 mV, *R*_2_ reaches as low as 8.4 Ω, while *C*_ϕ_ increases to 0.015 F, indicating improved proton coverage and enhanced H* spillover and OH* desorption. In contrast, both Mo and MoO_2_ counterparts exhibit higher *R*_ct_ values, indicating sluggish HER kinetics in alkaline media (Figure S9b,c). As the current density increases, high-speed imaging experiments prove that the small diameters of the H_2_ bubbles on Mo/MoO_2_ better leave catalytic surfaces and re-expose active sites compared to commercial Pt foil (Figure 3g), so Mo/MoO_2_ is proposed to have better HER performance, especially at high current density. To further understand this, contact angle (CA) measurements of a 1.0 M KOH droplet on the Mo/MoO_2_ and Pt foil surfaces (Figure 3h) reveal CAs of 66° and 14.5°, respectively. The HER performances of Mo/MoO_2_ and Pt foil at high current density can be assessed through the CAs of a 1.0 M KOH droplet on their surfaces, as seen in Figure 3h. The improved wettability of Mo/MoO_2_ due to its vertically oriented flake arrays with open channels/pores facilitates rapid H_2_ bubble release and enhances electrolyte transfer. Beyond the HER performance, the stability of electrochemical reactions is also a critical aspect for practical use. Long-term stability tests for the Mo/MoO_2_ catalyst are conducted using continuous cyclic voltammetry (CV), which demonstrated exceptional stability with no performance degradation in both electrolytes after 3000 cycles (Figure S10). Additionally, chronopotentiometry tests at a current density of 300 mA cm^–2^ confirmed steady HER performance over 100 h in both electrolytes (Figure 4e). Post-stability analysis via XRS (Figure S11) revealed no significant changes in the Mo/MoO_2_ catalyst after prolonged testing.

The overpotentials at 500 mA cm^–2^ and the Tafel slopes for Mo/MoO_2_ and Pt foil are presented in Figure 4a,b. It is clear that Pt shows a better HER performance in acidic than in alkaline media due to different proton supply rates, while the Mo/MoO_2_ shows decent and comparable HER activity in both media, indicating a similar local chemical environment despite the distinct bulk electrolytes. Furthermore, the rate-determining step (RDS) of the HER process varies between media, as illustrated by the Tafel plots in Figure 4b and Figure S12. Pt foil exhibits a Tafel slope as low as 34.5 mV dec^–1^ at pH 0, compared to 44.8 mV dec^–1^ at pH 14. Interestingly, the Tafel slope on Mo/MoO_2_ is only 38.2 mV dec^–1^ (in alkaline), demonstrating the transition of the RDS away from the proton recombination (Volmer–Tafel) or electrochemical desorption (Volmer–Heyrovsky) steps, even being comparable to the values of Pt foil and Mo/MoO_2_ (36.8 mV dec^–1^) in acidic environments. These findings indicate that a sufficient number of protons are present on the electrode surface for the HER process in both media, with Mo/MoO_2_ exhibiting acid-like HER behavior even in alkaline conditions. Moreover, we also explore the HER performance of the Mo/MoO_2_ in different alkaline (ranging from 0.01 to 2.0 M KOH) media (Figure 4a and Figure S13). It is clear that the HER performance of Mo/MoO_2_ improves significantly with higher OH^–^ concentrations. Further, the Tafel slope of Mo/MoO_2_ rises to ∼131 mV dec^–1^ when [OH^–^] reduces to 0.01 M KOH solutions, indicating that the RDS of the reaction is the first electron transfer. The H_2_O dissociation process involves an initial electron transfer to form adsorbed hydrogen and hydroxide ions. Therefore, in all alkaline media, H_2_O dissociation is usually the crucial step for proton generation in HER process.^36^ As [OH^–^] increases, the Volmer–Tafel or Volmer–Heyrovsky step becomes the RDS of the overall reaction. A large amount of OH^–^ can first be adsorbed on the cathode surface, which is beneficial for water desorption process to generate free H_3_O^+^ ions, indicating the formation of proton-concentrated cathode surface, leading to a local acid-like environment. We employ in situ Raman spectroscopy to further probe the local environment around the electrode in alkaline conditions. As shown in Figure 4c, the intensity of the characteristic peak at 740.8 cm^–1^, corresponding to the stretching vibrations of the O–W–O skeleton, significantly decreases as the applied voltage shifts from open circuit potential (OCP) to −0.3 V. Concurrently, the intensity of the ∼1256 cm^–1^ band, associated with the bending and stretching vibration modes of Mo–O–H species, increases.^37,38^ This observation confirms that the generation of hydrogen atoms covers the surface of MoO_2_ under a cathodic potential, where the H atoms are integrated into Brønsted acidic sites (H_*x*_MoO_*y*_ intermediates). Furthermore, the observed variations in 3233 and 3403 cm^–1^, which are attributed to trihedrally and tetrahedrally coordinated water at the interface, indicate that the incorporation of hydrogen is strongly related to interfacial water activation.^39,40^ As expected, Mo/MoO_2_ catalysts show progressively enhanced H signals with increasing applied overpotentials (from OCP to −0.3 V), suggesting the formation of a proton-concentrated surface on the Mo/MoO_2_ heterojunction at low overpotentials, thereby creating a local acid-like environment. Based on the above in situ spectroscopy and electrochemical analysis, the introduction of more electrons on the catalyst surface facilitates the reduction of H_3_O^+^ to H* at the active sites, which subsequently combine to form H_2_ gas (Figure 4d). Consequently, an acidic environment can be generated on the Mo/MoO_2_ surface under high [OH^–^] conditions, leading to acid-like HER kinetics.

**Figure 5:**
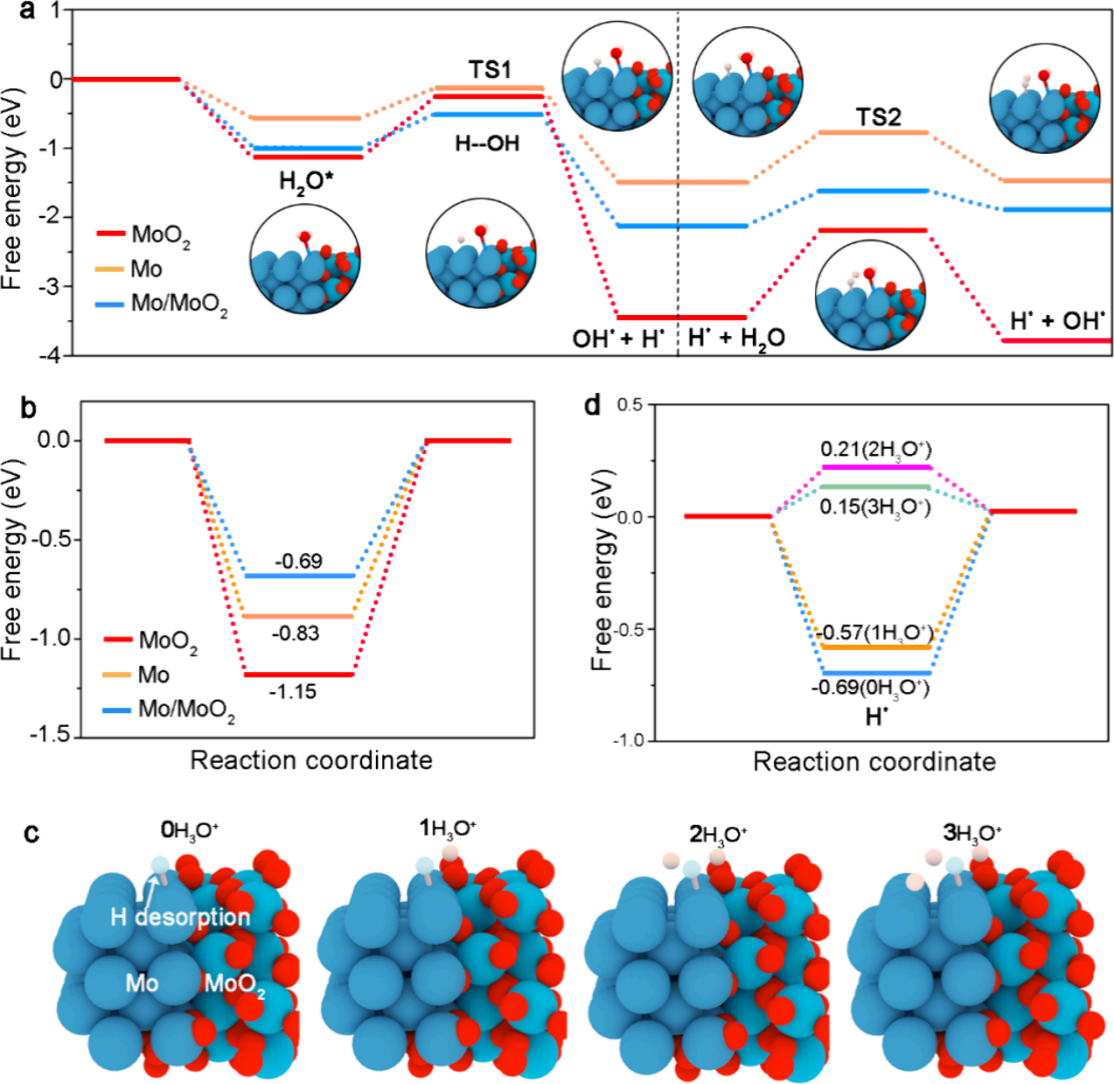
(a) Relative energy schematic of H_2_O dissociation on Mo/MoO_2_ interface, Mo, and MoO_2_ (TS: transition state). The accompanying calculated free energy diagrams for (b) H* desorption steps on the Mo/MoO_2_ interface, Mo, and MoO_2_, respectively. (c) Schematic diagram of H* (white) desorption models and (d) corresponding calculated free energies adjusted through (c) progressively increasing the concentration of neighboring H_3_O^+^.

To elucidate the substantial improvement of alkaline HER activity of Mo/MoO_2_ catalyst, DFT calculations are presented on model systems, with simulations of Mo and MoO_2_ models as references (Figure 5 and Figure S14). The results show that Mo/MoO_2_ (−0.94 eV) and MoO_2_ (−1.06 eV) exhibit more negative H_2_O adsorption free energies compared to Mo (−0.55 eV), indicating that both Mo/MoO_2_ and MoO_2_ surfaces are more favorable for the adsorption and activation of H_2_O reactants (Figure S14). As expected, MoO_2_ has a large kinetic energy barrier for the prior H_2_O dissociation step (Δ*G*_H_2_O_ = 0.81 eV), while this barrier is significantly reduced to 0.37 eV on the Mo/MoO_2_ surface (Figure 5a), suggesting that the oxygen-vacancy-rich Mo/MoO_2_ effectively facilitates H–OH bond cleavage. The ultralow energy barrier for H_2_O dissociation on the Mo/MoO_2_ catalyst surface facilitates the generation of protons; the subsequent proton separation can dictate the reaction rate of the alkaline HER process. The hydrogen adsorption free energy (Δ*G*_H*_) of the H* intermediate serves as an effective descriptor for gauging the activity of HER, where the ideal Δ*G*_H*_ is often close to the thermoneutral value of zero. As shown in Figure 5b, the Δ*G*_H*_ values on pure Mo and MoO_2_ are as high as −1.15 and 0.83 eV, respectively. Accordingly, the obtained Δ*G*_H*_ decreases sharply to −0.69 eV when Mo and MoO_2_ are combined as heterojunctions, indicating a suitable hydrogen adsorption capability on the Mo/MoO_2_ catalyst surface. Encouragingly, the Δ*G*_H*_ value (0.15 eV) of Mo/MoO_2_ significantly approached the ideal thermoneutral value after the introduction of H_3_O^+^ intermediates at the Mo/MoO_2_ interface, which suggests the hydrogen desorption kinetics on the Mo/MoO_2_ catalyst surface can be effectively optimized by increasing adjacent H_3_O^+^ concentration (Figure 5c,d).

## 3 Conclusion

In summary, we successfully developed a large-area self-standing Mo/MoO_2_ catalytic electrode with a vertically oriented porous flake array structure by a simple and scalable pyrolysis-reduction method, which performs well for alkaline HER, especially at high current density. This is due to the deliberately engineered local acid-like environment, achieved by adjusting proton coverage on the Mo/MoO_2_ catalyst surface during alkaline HER. The designed Mo/MoO_2_ heterojunction follows the acid-like HER kinetics with an ultralow overpotential of 65 mV at −10 mA cm^–2^ and a small Tafel slope of −38.2 mV dec^–1^ as well as a long-term electrocatalytic durability of over 60 h at a high current density of 300 mA cm^–2^. Combined with in situ Raman spectroscopy characterizations and theoretical analysis, we show that heterojunction engineering of Mo and MoO_2_ enrich local proton concentration on Mo/MoO_2_ surface under low overpotentials facilitating the thermodynamically favorable Volmer–Tafel pathway and resulting in accelerated HER kinetics. Moreover, the high HER performance and durability of Mo/MoO_2_ at high current density are attributed to the bubble fining sieve structure of porous flake arrays with superior bubble desorption and water adsorption. This pyrolysis-reduction reaction is facile for the massive synthesis of porous flake arrays catalysts with superior acid-like HER performance and, through delicate design, provides a practical route to produce more advanced catalysts toward improving HER performance in alkaline electrolyte.

## 4 Experimental Section

### Growth of MoO_3_ Prisms

Prism-shaped MoO_3_ crystals are grown directly on the Mo plate by a traditional oxidizing roasting method by heating treatment. The cheap industrial-grade Mo plate (2 cm × 2 cm) is treated by diluted HCl solutions, followed by washing with acetone, ethanol, and distilled water, followed by drying at 60 °C. The pretreated Mo plate is placed into an alumina boat and positioned at the center of a quartz tube furnace. The furnace is heated to 650 °C at a rate of 10 °C min^–1^ and maintained at this temperature for 120 min in an air atmosphere. The self-standing MoO_3_/Mo plate is obtained in a quartz tube furnace.

### Growth of MoO_*x*_ Flake Arrays

In a typical procedure, pyrolysis at 800 °C under an Ar atmosphere converted the MoO_3_ prisms into MoO_*x*_ flake arrays by using a chemical vapor deposition (CVD) system. The self-standing MoO_3_/Mo plate (2 cm × 2 cm) as the source is positioned within an aluminum oxide boat at the middle of furnace. First, high-purity Ar is introduced into the tube at a flow rate of 200 sccm for 30 min, creating a stable environment devoid of oxygen in the furnace. Subsequently, the flow rate of Ar is maintained at 100 sccm, and the furnace temperature is increased to the growth temperature of 800 °C at a rate of 40 °C min^–1^. The temperature is maintained for 30 min, after which it is gradually reduced to 300 °C. At this point, the furnace hood is opened to quickly cool the tube to room temperature. The entire pyrolysis-reduction procedure is performed at standard atmospheric pressure.

### Growth of Mo/MoO_2_ Metallic Heterojunction

The self-standing Mo/MoO_2_ plate is produced through subsequent thermal treatment. The desired Mo/MoO_2_ metallic heterojunction are obtained by a controlled thermal reduction reaction of MoO_*x*_ flake arrays at 700 °C in an Ar/H_2_ (v/v = 10:1) atmosphere for 30 min. In contrast, self-standing Mo samples are synthesized at higher temperatures of 800, 900, or 1000 °C under the same Ar/H_2_ (v/v = 10:1) atmosphere for 30 min.

### Electrochemical Measurement

All electrochemical tests are carried out at room temperature. The HER electrochemical experiments employ a three-electrode setup on a CHI 660E workstation. For these tests, a graphite rod serves as the counter electrode, while a standard Hg/HgO electrode is employed as the reference electrode. The self-standing Mo/MoO_2_ catalytic electrode (0.5 × 1.0 cm^2^, area immersed in electrolyte is 0.5 × 0.5 cm^2^) is used as the working electrodes. To prepare the Mo, MoO_2_, and Mo/MoO_2_ electrode slurry, 5 mg of the powder catalysts is mixed with 1 mL of ethanol and 80 μL of 5 wt % Nafion, followed by sonication for 30 min for fabrication of the homogeneous catalyst ink. 0.25 mL of slurry is dropped onto the polished glassy carbon rotating disk electrode and then dried at room temperature in a vacuum oven. In comparison, the self-standing electrode of the physical mixture of Mo and MoO_2_ powders is prepared using the same preparation procedure. All the potentials are referenced to an RHE by adding 0.923 V (0.099 + 0.059 × pH) in a 1.0 M KOH aqueous solution. The HER polarization curves are obtained using LSV at a sweep rate of 5 mV s^–1^ in H_2_-saturated 1.0 M KOH solution. Meanwhile, the long-term stability tests are assessed through continuous cyclic voltammetry (CV) at a scan rate of 200 mV s^–1^ in 1.0 M KOH. CV curves are collected at different scan rates in the potential range of 0.1–0.2 V vs RHE to evaluate the double-layer capacitance values for HER. EIS spectra are measured over a frequency range of 106–0.01 Hz with a 10 mV amplitude at specific potentials. All data are collected with an *iR* correction.

## Supporting Information Available

Schematic illustration of the preparation process; characterization of the catalysts included OM, SEM, XRD, TEM, and EDS; CV curves at different scan rates; EIS measurements; LSV curves before and after 3000 CVs, and XPS spectra after HER stability tests; LSV curves and corresponding Tafel plots under different media; and calculated free energies (PDF)
