# Supplementary material for: Self-Standing Mo/MoO2 Porous Flake Arrays for Efficient Hydrogen Evolution Reaction in High-pH Media
Source: ACS Appl Mater Interfaces. 2024 Oct 7;16(41):55569–77. doi: 10.1021/acsami.4c14140 (PMC11492176; doi:10.1021/acsami.4c14140)
Supplement: Supplementary file 1 — am4c14140_si_001.pdf [file am4c14140_si_001.pdf]

Supporting Information

The self-standing Mo/MoO_2 porous flake arrays for efficient hydrogen evolution reaction in high-pH media

Chuanyong Jian[1], Jiashuai Yuan[1,2], Qian Cai[1], Wenting Hong[1], Wei Liu[1*]

[1]CAS Key Laboratory of Design and Assembly of Functional Nanostructures, Fujian Provincial Key Laboratory of Nanomaterials, Fujian Institute of Research on the Structure of Matter, Chinese Academy of Sciences, Fuzhou, 350002, China

[2]College of Chemistry and materials, Fujian Normal University, Fuzhou, Fujian, 350007, China

E-mail: liuw@fjirsm.ac.cn

Structural characterizations

X-ray diffraction (XRD) spectra is recorded on the D8 ADVANCE diffractometer with Cu Kα radiation (λ = 1.5418 Å). Transmission electron microscopy (TEM), high-resolution TEM (HRTEM), and energy dispersive X-ray spectroscopy (EDS) mapping studies are performed using a probe-corrected transmission electron microscope operating at 200 kV (FEI Titan F20 TEM). Scanning electron microscopy (HITACHI UHR FE-SEM SU8010) is employed to characterize the morphologies of the as-grown samples. X-ray photoelectron spectroscopy (XPS) measurements are performed by a ESCALAB 250Xi system (Thermo Fisher), equipped with a 100 W Al Kα source on a spot size of 100 μm at a 45° incident angle. The binding energy scale is calibrated with the C 1s peak at 284.8 eV. Raman spectra are collected using a Raman microscopy system (LabRAM HR) equipped with a 532 nm laser.

Theoretical Calculations

Density functional theory calculations were carried out in Atomistix Tool Kit 2024.[1] with the Perdew-Burke-Ernzerhof exchange-correlation within the generalized gradient approximation. A 4 × 4 × 1 Monkhorst-Pack k-point grid with the cutoff energy of 85 Hartree was employed. The Pulaymixer algorithm controlled the self-consistent iterations with 0.0002 Ry tolerance and 100 maximum steps. The structures were optimized to a maximum force of 0.05 eV $Å^{-1}$ and the maximum stress of 0.005 eV $Å^{-3}$ with a limited memory Broyden–Fletcher–Goldfarb-Shanno algorithm.[2] Based on these conditions, the corresponding optimization of M (Mo, MoO_2, Mo/MoO_2) slabs was constructed with a vacuum region of 15 Å on z-direction. The differential binding energy is used to describe the stability of hydrogen, defined as

$$\Delta E_H = E\,(M + nH) - E(M + (n\text{-}1)H - 1/2E(H_2)$$

where E (M + nH) is the total energy for the M slab adsorbed n hydrogen atoms, E(M + (n-1)H) is the total energy for (n-1) adsorbed hydrogen atoms on the M slab, and $E(H_2)$ is the energy of a gas phase hydrogen molecule. The Gibbs free energy ΔG_H for hydrogen adsorption can be calculated as

$$\Delta G_H = \Delta E_H + \Delta ZPE - T\Delta S_H$$

where ΔZPE is the zero-point energy difference between the adsorbed state of the system and the gas phase state, ΔS_H is the entropy difference between the adsorbed state of the system and the gas phase standard state (300 K, 1 bar).

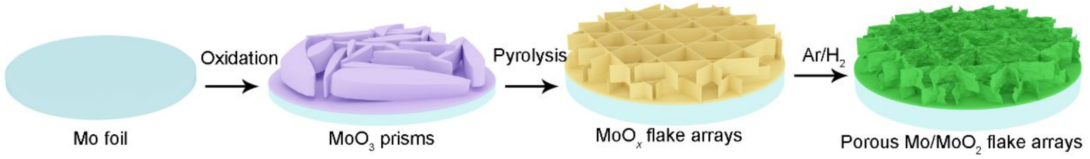

Figure S1. Schematic illustration of the preparation process of large-area the self-standing Mo/MoO_2 electrode.

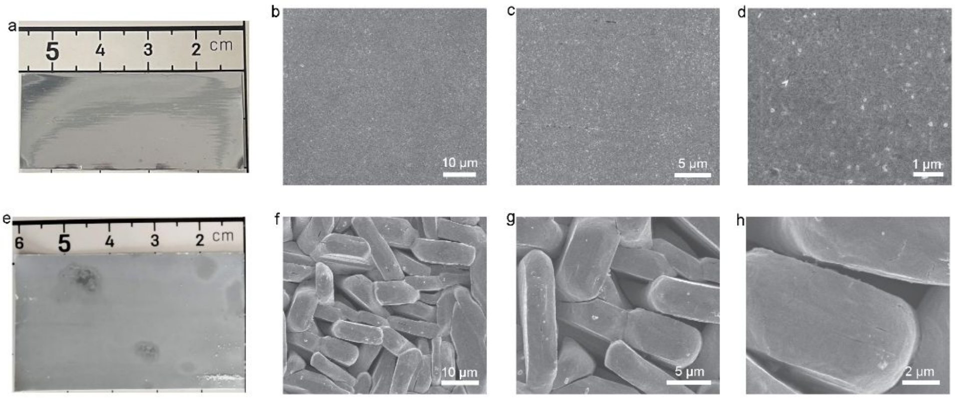

Figure S2. (a, e) Optical images of the as-prepared Mo and MoO_3 electrodes. Typical scanning electron microscopy (SEM) image of **(b-d)** Mo plate and **(f-h)** MoO_3 prisms.

As shown in Figure S2, the prism-like MoO_3 crystals with the thickness of about 2.5~5 μm are uniformly in situ grown on smooth Mo plate surface (Figure S2a-d) via a controlled oxidation reaction in the air at 650 ˚C. A magnified SEM images clearly show that the prism-like MoO_3 crystals exhibit a smooth surface (Figure S2f-h).

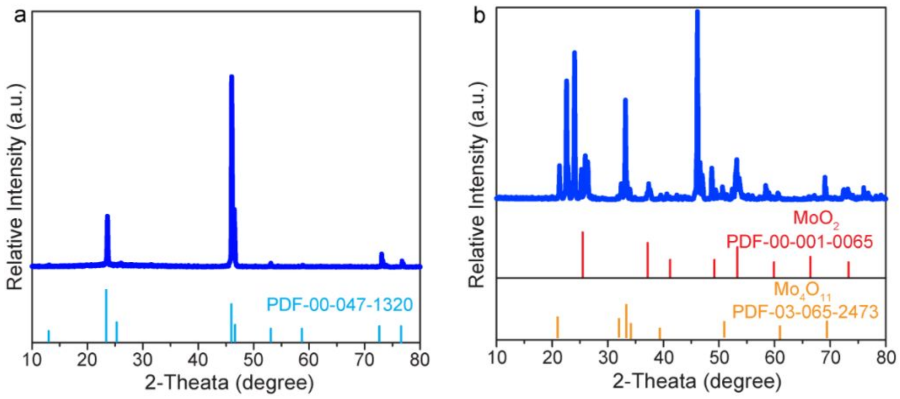

Figure S3. (a) The XRD pattern of the MoO_3 when the Mo plate is heated at 650 ºC in air. **(b)** The XRD pattern of the MoO_x flakes (Mo_4O_{11} + MoO_2) when the MoO_3 are heated for 1 h at 700 ºC in Ar atmosphere.

The product of the Mo plate calcined at 650 ºC for 2 h in air is surveyed with X-ray diffraction using Cu-Ka radiation. In Fig. S3a, the sharp X-ray diffraction diffraction peaks at 23.5º, 46.0º, 46.6º, 73º and 76.7º are indexed to MoO_3 (PDF-00-047-1320). In Fig. S3b, the sharp X-ray diffraction diffraction peaks at 21.4º, 32.4º, 33.2º, 50.6º, 60.6º and 68.7º originate from the Mo_4O_{11} (PDF-03-065-2473). The peaks located at 26.3º, 37.0º, 41.5º, 49.5º, 53.7º, 60.5º and 66.9º are indexed to metallic MoO_2 (PDF-00-001-0065). The XRD analysis confirms that the obtained the MoO_x flakes consists of Mo_4O_{11} and MoO_2.

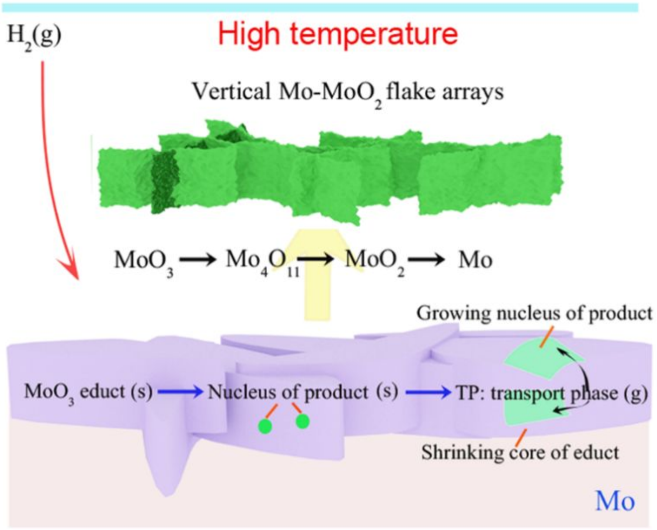

Figure S4. Schematic illustration of thermal redox reaction.

The schematic analysis of different stage of reduction is shown in Figure S4. The initial stage of reduction is nucleation and growth of grains forming on the surface of the educt particle. Due to the intentional introduction of reaction gas, some cracks and fissures are generated from the surface to center. This behavior extent follows the cracking core model (CCM). Meanwhile, the extent of reaction of the second stage develops according to the shrinking core model (SCM). As the product phase grows, the shrinkage of the unreacted core gradually produces the porous product layer.[1-3]

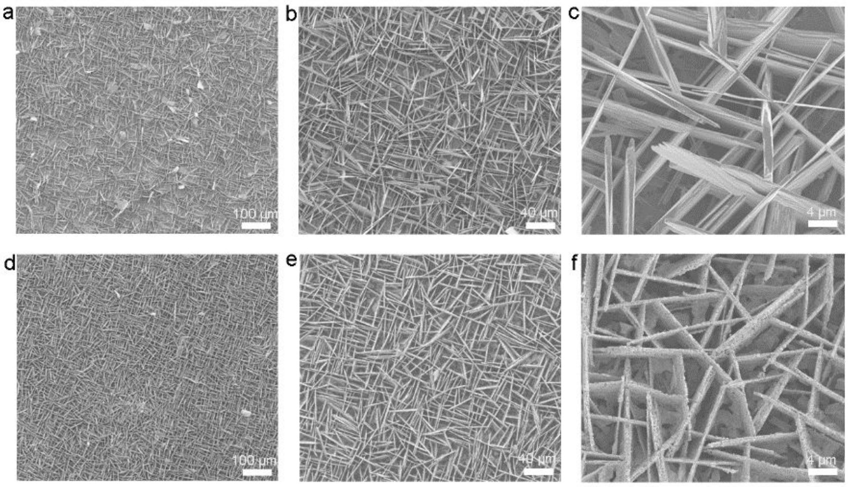

Figure S5. SEM images of **(a-c)** MoO_x and **(d-f)** Mo/MoO_2 flake arrays.

As shown in Figure S5a-c, dense vertically oriented flake arrays with with an average thickness of 1-2 μm as well as a height and bottom length of 3-5 and 10-20 μm are in-situ grown on the Mo plate surface. After the annealing of MoO_x flakes in Ar/H_2 atmosphere, these smooth flakes are transformed into porous structure with some large channels and small nanopores (Figure S5d-f). Particularly, the $Mo/MoO_2/Mo$ substrate is suitable employed as a current collector due to its high surface area, commercial availability and good conductivity. The unique three-dimensional (3D) porous flake arrays structure is benefit to enhance mass transport properties and expose large number of catalytically active sites for catalytic reaction.

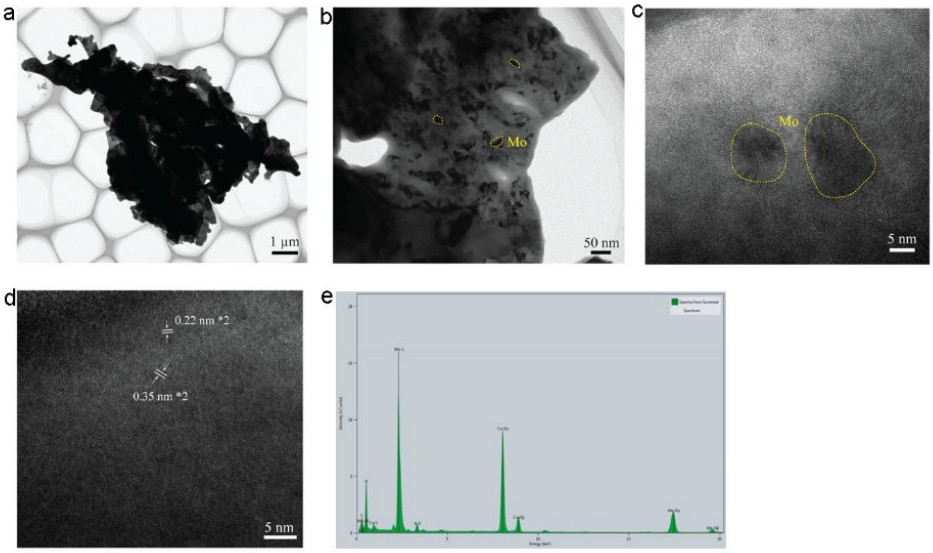

Figure S6. (a, b) Low-magnification TEM images, **(c, d)** High-resolution TEM images, **(e)** EDS spectrum of Mo/MoO_2.

From low-magnification TEM images, Mo/MoO_2 samples inherit the original MoO_x flakes-like morphology (Figure S6a, b). The Mo/MoO_2 exhibits clear lattice fringes with interplanar distances of 0.22 and 0.35 nm, corresponding to the (200) and (-111) facet of Mo NPs and underlying MoO_2 matrix, respectively.

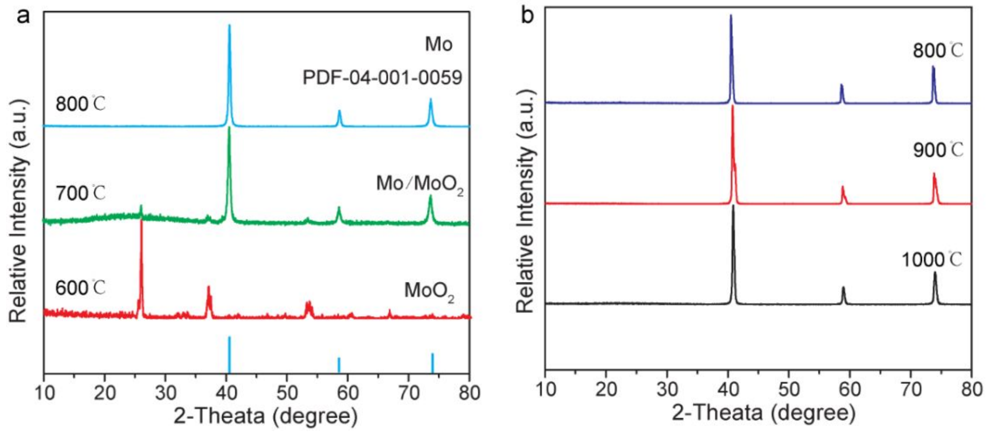

Figure S7. (a) The XRD pattern of the MoO_2, Mo/MoO_2 and Mo. **(b)** The XRD pattern of the Mo when the the MoO_x flakes are heated at 800 ºC, 900 ºC and 1000 ºC in under a mixture of Ar/H_2 (v/v = 10:1) atmosphere.

When the as-synthesized MoO_x flakes are calcined in a mixture of Ar/H_2 (v/v = 10:1) atmosphere at 700 ºC, the XRD patterns of featured characteristic peaks of MoO_2 phases at 26.3°, 37.0º and 53.7°, and three peaks of metallic Mo at 40.1°, 59.0º and 74.1º. The successful growth of metallic Mo is again confirmed by XRD analysis in Figure S7a. As shown in Fig. S7b, the sample only exhibits three peaks of metallic Mo after high-temperature pyrolysis treatment (above 800 º C).

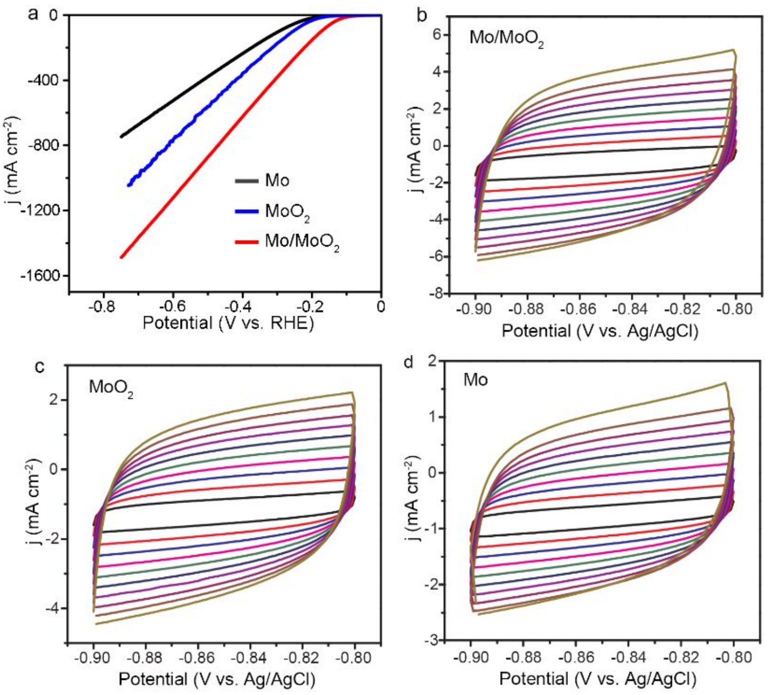

Figure S8. **(a)** The polarization (LSV) curves of MoO_2, Mo and Mo/MoO_2 catalysts in 1.0 M KOH electrolyte using the rotating disk electrode technique at 1600 rpm. Cyclic voltammetry (CV) measurements at different scan rates. **(b)** Mo/MoO_2, **(c)** MoO_2, and **(d)** Mo in the voltage window from -0.9 to -0.8 V vs. Hg/HgO at different scan rates (10-100 mV s^{-1}) in 1.0 M KOH electrolyte.

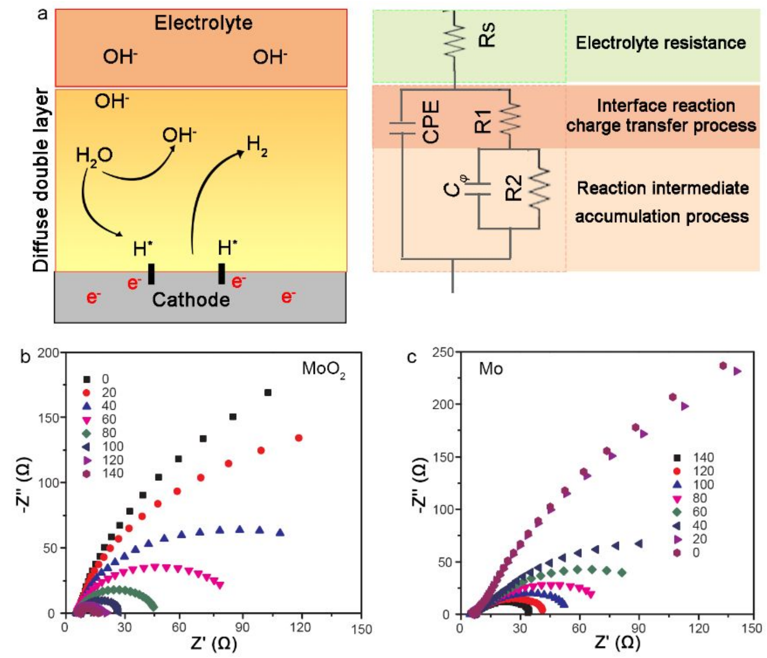

Figure S9. (a) Equivalent circuit model used in the fitting of operando impedance data. Electrochemical impedance spectra (EIS) measurements of Mo and MoO_2 catalysts. Nyquist plots of **(b)** MoO_2 and **(c)** Mo.

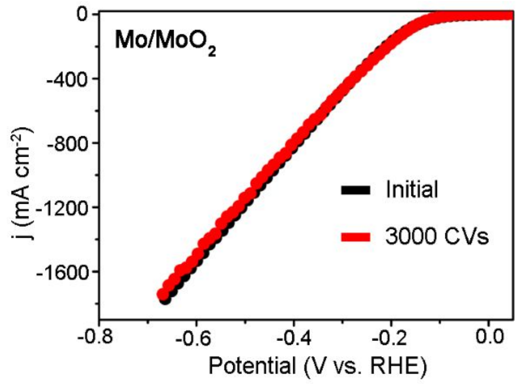

Figure S10. LSV curves of Mo/MoO_2 catalyst before and after 3000 CVs.

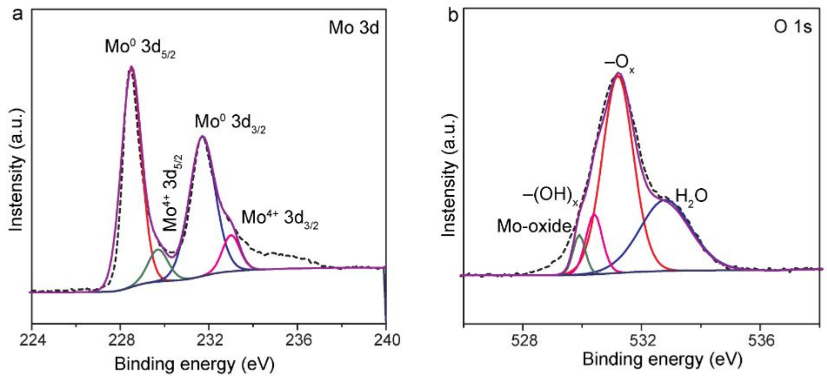

Figure S11. XPS spectra of Mo/MoO_2 after stability test. **(a)** Mo 3d spectra, (**b)** O 1s XPS spectra.

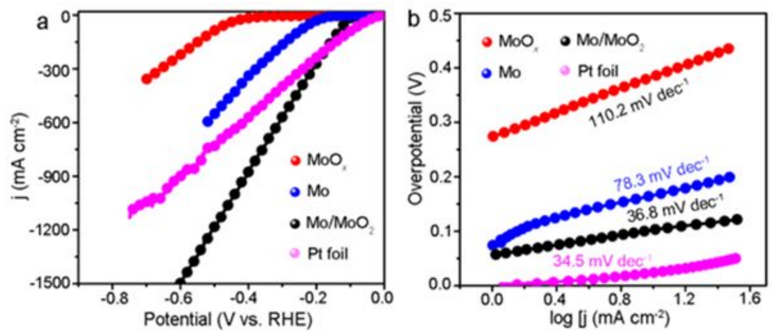

Figure S12. (a) The polarization (LSV) curves of MoO_x, Mo, Mo/MoO_2, and Pt foil catalysts in 0.5 M H_2SO_4 solution. **(b)** The corresponding Tafel plots.

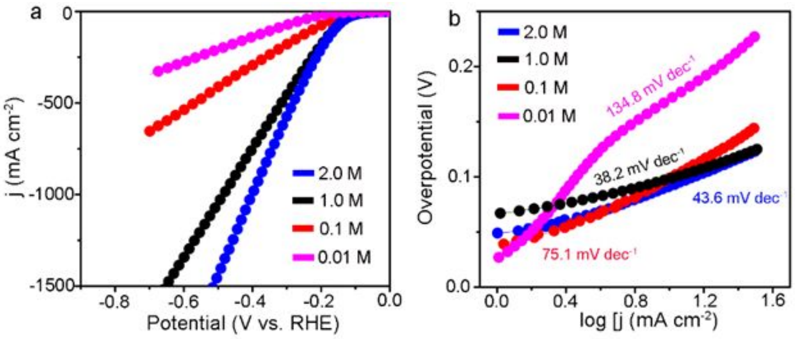

Figure S13. (a) The polarization (LSV) curves of Mo/MoO_2 under different alkaline media. **(b)** The corresponding Tafel plots.

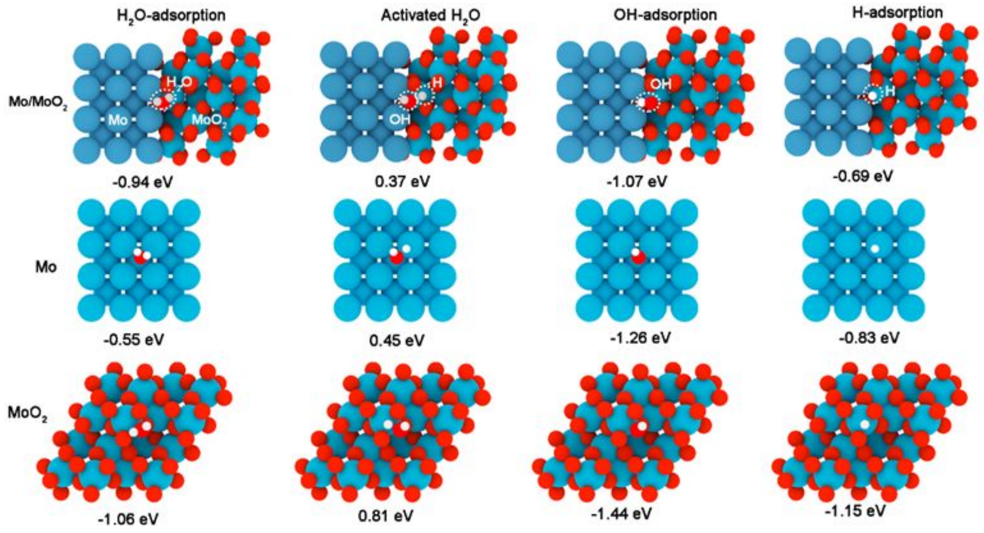

Figure S14. Calculated free energies of H_2O-adsorption, activated H_2O, OH-adsorption and H-adsorption. Blue balls: Mo; red balls: O; white balls: H.

Table S1. Comparison of overpotentials (10 mA cm^{-2}) and Tafel slopes of Mo/MoO_2 catalyst and previously reported excellent transition-metal-oxide based HER catalysts in alkaline solutions.

Catalysts	Overpotential (η10, mA cm^{-2})	Tafel slope (mV dec^{-1})	Reference
**Mo/MoO_2**	**-65**	**38.2**	**This work**
$mMoO_3$	-138	-56	*Adv. Energy Mater.*, 2016, 6, 1600528
O_v-α-MoO_3	-138	-60	*J. Mater. Chem. A*, 2019, 7, 257-268
GDY/MoO_3	-170	-70	*J. Am. Chem. Soc.*, 2021, 143, 8720-8730
Co-$WO_{2.7\text{-}x}$	-59	-86	*Chem. Eng. J.*, 2023, 451, 138939
Ni-MoO_2/NF	-49	-75.1	*Appl. Catal. B Environ.*, 2022, 301, 120818
$Co_2Mo_3O_8$	-37	-58	*Nano Energy*, 2021, 87, 106217
NiO/Ni	-121	-88	*Nano Energy*, 2017, 35, 207-214
MoS_2/Ni_2O_3H	-84	-82.3	*Small*, 2020, 16, 2002212
Co@NCNT/$CoMo_yO_x$	-94	-76	*J. Mater. Chem.A*, 2022, 10, 3953-3962
$Ni(OH)_2/MoS_2$	-80	-60	*Nano Energy*, 2017, 37, 74-80
CuCo/$CuCoO_x$	-115	-55	*Adv. Funct. Mater.*, 2018, 28, 1704447

Table S2. The fitted parameters of EIS data from Mo/MoO_2 catalyst treated by increasing applied overpotentials. CPEs (CPE-T and CPE-P) are routinely used in place of pure capacitors to model this interfacial layer.

η (mV)	$R_s(\Omega)$	$R_1(\Omega)$	$R_2(\Omega)$	C_φ (F)
0	2.8	6.3	864.9	0.012
-20	2.7	6.3	35.2	0.012
-40	2.6	3.1	16.7	0.014
-60	2.7	3.1	8.4	0.015
-80	2.6	3.1	2.3	0.017
-100	2.6	3.1	1.2	0.018

References

[1] Park, J. Y.; Levenspiel, O., The crackling core model for the reaction of solid particles. *Chem. Eng. Sci.* **1975,** *30*, 1207-1214.

[2] Schulmeyer, W. V., Ortner, H. M., Mechanisms of the hydrogen reduction of molybdenum oxides. *Int. J. Ref. Met. H.* **2002,** *20*, 261-269.

[3] Spevack, P. A.; McIntyre, N. S., Thermal reduction of molybdenum trioxide. *J. Phys. Chem.* **1992,** *96*, 9029-9035.
